# Nutritional and Physicochemical Quality of Vacuum-Fried Mango Chips Is Affected by Ripening Stage, Frying Temperature, and Time

**DOI:** 10.3389/fnut.2020.00095

**Published:** 2020-07-17

**Authors:** Fitriyono Ayustaningwarno, Elise van Ginkel, Joana Vitorino, Matthijs Dekker, Vincenzo Fogliano, Ruud Verkerk

**Affiliations:** ^1^Food Quality and Design, Wageningen University & Research, Wageningen, Netherlands; ^2^Department of Nutrition Science, Faculty of Medicine, Diponegoro University, Semarang, Indonesia; ^3^Center of Nutrition Research, Diponegoro University, Semarang, Indonesia

**Keywords:** vacuum frying, mango, ripening, vitamin C, β-carotene, fat, color, texture

## Abstract

For the production of healthier fruit snacks, vacuum frying is a promising alternative for atmospheric frying, to reduce the oil content, while maintaining a high nutritional quality. This paper evaluates the effect of ripening stages, frying temperature, and time on the quality of vacuum-fried mango. Unripe mango was dehydrated faster than ripe mango and had a higher hardness after frying at 110 and 120°C. Fat content in fried ripe mango was higher. Total ascorbic acid and β-carotene in both ripening stages were not different, but after frying total ascorbic acid in unripe mango remains higher. A novel image analysis was applied to quantify the color distribution of fried mango. Color changes in unripe mango were more susceptible to temperature and time. Considering all quality parameters, vacuum frying of unripe mango at the optimal condition of 100°C for 20 min is preferred for producing high-quality healthier fruit snacks.

## Introduction

Consumers have a strong desire for fried food products because of their unique flavor–texture combination. However, the increased awareness of consumers toward the relationship between food, nutrition, and health stimulates the food industry to use alternative processing methods complying the demand for healthier snacks. In this paper, we study an alternative frying process to meet these demands by reducing the oil uptake and maintaining a high nutritional value.

Technically deep-frying is heating and dehydrating foods with associated oil uptake by immersing them in an edible fat at 165–190°C (1). Compared to other cooking methods, deep-frying generates products with a unique color, texture, and flavor.

Deep-frying dries the product, giving it a crust and making it crispy (2). However, excessive oil absorption is an undesired side effect of the process that could be limited by various strategies such as dripping and post-frying centrifugation (3). Additionally, a high frying temperature might reduce the content of nutrients present in the raw material (4). In the framework of the increasing demand for healthier snack choices, industries are developing low-fat alternatives. Techniques such as drying, extrusion, and baking have not always been able to satisfactorily meet the sensory characteristics of fried foods (5). Consumers do not want to compromise on organoleptic properties in exchange for healthier products (6). Altogether, vacuum frying might be a promising technology for healthier fried products.

Vacuum frying is similar to atmospheric frying but is carried out under reduced pressure below 10 kPa, causing a decrease in the boiling point of water in the fried products (4). Therefore, the frying temperature can be reduced to as low as 90°C, which allows preservation of the food nutritional characteristics, flavor, and aroma. This is of particular relevance for fruits which contain high amounts of thermolabile vitamins and phytochemicals. In this respect, characteristics of the fruit matrix can play a very important role and is an underexposed factor in the scientific literature up to now (7).

Several studies on vacuum-fried food have been done for pineapple (8) and apple (9). The effect of ripening on the physicochemical quality of vacuum-fried fruit has already been investigated for banana and jackfruit. Yamsaengsung and Ariyapuchai (10) found that vacuum-fried ripe banana has a higher volume expansion than unripe banana, even though there was no difference between them in overall sensory acceptability. Diamante (11) found that vacuum-fried ripe jackfruit has a higher moisture and fat content, while it was more yellow and less crunchy, has more aroma, and is sweeter than half-ripe jackfruit.

Studies on vacuum-fried mango have been done to compare the vacuum frying technique to atmospheric frying on the oil content, color, texture, total carotenoid content, and the like (12) and to study the effect of osmotic dehydration on moisture and oil content, expansion, density porosity, color, texture, and total carotenoid and sensory characteristics (13). However, the effect of ripening stage on the quality of vacuum-fried mango has not been studied before.

As a climacteric fruit, mango quality is strongly influenced by ripening. During ripening, physiological, biochemical, and molecular changes are initiated in the mango matrix by the autocatalytic production of ethylene and increase in respiration rate. Some of these changes include increased biosynthesis of carotenoids, a decrease in ascorbic acid, conversion of starch into sugars, and the softening of the fruit promoted by the pectinase action on the cell wall. Another influenced quality attribute is color. Color changes in mango result from carotenoid accumulation in the pulp (14).

The objective of this study was to investigate the effect of ripening stages, frying temperature, and time on the nutritional and physicochemical quality of vacuum-fried mango. The physicochemical quality was characterized by measuring the key parameters moisture and fat content, color and texture. While the nutritional value was assessed by analyzing vitamin C and β-carotene content as key parameters for, respectively, water- and fat-soluble nutrients in mango. The research hypothesis was that quality of vacuum-fried mango chips is affected by ripening stage, frying temperature, and time.

## Materials and Methods

### Raw Material

Unripe (stage 2, firmness 68.4–87.9 kg/m^2^) and ripe (stage 4, firmness 24.4–39.1 kg/m^2^) mango (*Mangifera indica* L. cv. Kent) from Brazil were supplied by Bakker Barendrecht B.V. (The Netherlands) and stored at 11°C and used within 5 days after arrival. Just prior to the frying experiment, the mangoes were selected based on ripeness indicators, including total soluble solids (TSS), to indicate the sugar content and firmness (15) as shown in Table 1. In order to study the role of the physiological maturity, stages 2 and 4 were selected to represent the unripe and ripe mango with enough internal matrix differences, while still having suitable properties for handling the fruit.

**Table 1:** Initial values for firmness and total soluble solid content of unripe and ripe mangoes used for vacuum-frying experiments.

**Ripeness**	**Firmness (kg/m^**2**^)**	**TSS (^**°**^Brix)**
**Unripe**	78.25 ± 0.44^a^	16.03 ± 0.08^a^
**Ripe**	31.91 ± 0.26^b^	16.41 ± 0.11^b^

*Different superscripts at firmness and TSS values show significant difference (p ≤ 0.01) between ripe and unripe mango N = 423*.

Firmness was measured using fruit penetrometer FT327, equipped with an 8-mm tip (Nieuwkoop B.V., the Netherlands). The firmness measurements were done on each peeled mango cheek with three repetitions and were expressed in kg/m^2^. TSS content was measured three times from juice obtained from mango cheek using a refractometer (HI96801, Hanna Instruments) and was expressed in °Brix. After selection, mangoes were peeled, and the seed was removed and halved. The halved fruits were cut into 4-mm-thick slices with a mandolin (V5 Power, Börner, Germany) to ensure the fast heat penetration to the center of the chips but not collapse during the processing.

### Vacuum Frying Procedure

Mango slices were vacuum fried in 2-kg batches using a pilot scale industrial vacuum fryer (Florigo Industry B.V., The Netherlands) containing 250 L fresh high oleic sunflower oil. A high oil-to-fruit ratio was needed to diminish temperature drop after the fruit was submerged into the oil. The vacuum fryer was equipped by an automatic basket rotator, two heat exchangers to cool and heat the oil, and an atmospheric spinner to remove surface oil. Some pilot experiments have been carried out to determine the optimal conditions for thickness of mango slices and vacuum frying pressure. Based on these results, 4-mm mango slices were fried at 10 kPa at times and temperatures as listed in Table 2. This working pressure produces water boiling at 44.3°C.

**Table 2:** Settings used for vacuum frying mango chips.

**Oil temperature (^**°**^C)**	**Frying time (minutes)**					
90	5	10	15	25	35	50
100	5	10	15	20	27.5	35
110	2.5	5	10	15	20	25
120	2.5	5	7.5	10	12.5	15

Two samples of mango slices (1 kg of each of the two ripening stages) were loaded into the vacuum chamber. After 60 s, the desired vacuum pressure was reached, and the basket was submersed into the oil to initiate the frying time. During frying, the basket was rotated back and forth at 17 rpm for 60 s to ensure that the heat and oil were evenly distributed. To stabilize the temperature, a heat exchanger to heat and cool the oil was used. Once the frying was finished, the basket was lifted from the oil and shaken for 20 s inside the vacuum chamber to remove excess of oil. The vacuum chamber was then pressurized, and the vacuum-fried mango was centrifuged to remove surface oil for 60 s at 100 g (MSD-500HD, Eillert B.V., the Netherlands). Then, mango chips were packed in sealed plastic bags and stored at −20°C until further use. All frying experiments were performed in duplicate.

### Moisture and Fat Content

Moisture content of the samples was determined in triplicate per frying experiment with a forced convection oven at 100°C until constant weight and described in % fresh weight. Fat content of the fried mango chips was determined in duplicate per frying experiment with the Soxhlet method using 200 ml petroleum ether 40–60°C after drying overnight and then described in % dry basis (db) (16).

### Texture

Texture of the mango chips was measured with a texture analyzer (TA.XT.Plus, Stable Micro Systems, UK) using a three-point bending test according to Da Silva and Moreira (17) with some modifications. One mango chip was placed on two parallel edges (16 mm wide), and the probe selected was a 1-mm-thick steel blade. Settings used were as follows: 0.50 mm/s test speed, 10.00 mm/s post-test speed, 15 mm distance, and 5 kg load cell. Results from ten replications per frying experiment were expressed as hardness in N.

### Total Ascorbic Acid

Since ascorbic acid (AA) easily oxidized into L-dehydroascorbic acid (DHA), vitamin C was calculated as total ascorbic acid (TAA) which sums of AA and DHA. AA was reduced into DHA using TCEP (tris-2-carboxyethyl phosphine) and then calculated together into TAA. The extraction and HPLC analysis was conducted according to the methods of Hernández, Lobo (18), and Wechtersbach and Cigić (19) with modification. Two grams of sample was used and homogenized with 25 ml metaphosphoric acid-tert-butylhydroquinone (MPA-TBHQ) solution using Ultra Turrax at high speed for 45 s and was centrifuged for 20 min at 4,000 rpm at 4°C. The supernatant was then centrifuged again in a 5-ml preweighed reaction tube at 10,500 rpm for 20 min at 4°C. An amount of 1.485 ml sample was added by 15 μl TCEP, filtered using a 0.2-μm CA filter to an amber vial and ready to be injected into the HPLC. Three measurements per frying experiment were conducted, and the values are expressed in mg TAA/100 g of dry basis (db). A calibration curve was prepared using 10 mg/ml ascorbic acid in MPA TBHQ, filtered through a 0.2-μm CA filter, and diluted in 8 steps to get a range from 200 μg/ml to 1.56 μg/ml with *R*^2^ = 0.997. HPLC analysis was done using thermo separation products Spectra Series HPLC with a binary gradient pump and UV detector at 245 nm and Polaris 5 C18 A 150 × 4.6 mm 5 μm column. The twenty-μl sample was injected using orthophosphoric acid 0.2% in Milli Q water as mobile phase, with a 5.5-min run time at a 1.0-ml/min flow.

### β-Carotene

The extraction and HPLC analysis of β-carotene was conducted according the methods of Salur-Can, Türkyilmaz (20), with modification. During extraction, 1 g sample was used and dissolved into 1.5 ml of Milli Q water and 7 ml hexane, then the samples were homogenized using Ultra Turrax and centrifuged 5 min for 3,000 rpm to get the supernatant; the pellets were extracted again two times, on a third time using 10 ml tetrahydrofuran (THF) instead of hexane. The orange upper liquid then taken out (from hexane and THF extraction), the solvents were evaporated using vacuum evaporator at 40°C, 270 mbar vacuum. The extract then dissolved into 2 ml buffer MeOH-THF 1:1 + 0.01% BHT, filtered through a 0.2-μm filter, ready to be injected into HPLC. Three measurements per frying experiment were conducted, and the values are expressed in μg/g dry basis (db) of β-carotene. A calibration curve was prepared using 0.1 mg/ml β-carotene (Sigma Aldrich, 22040) in THF including 0.1% BHT, filtered through a 0.2-μm filter, and diluted in 6 steps to get a range from 50 to 0.78 μg/ml with *R*^2^ = 0.999. HPLC analysis was done using HPLC with a Dionex Ultimate 3000 RS equipped with Phenomenex Onyx monolithic C18 column (100 × 4.6 mm, pore size of 130 Å). Twenty μl of sample was injected with 60% acetonitrile (ACN), 30% MeOH, 10% ethyl acetate (ETAC), and 0.1% trimethylamine (TEA) mobile phase, with a run time of 10 min at flow rate of 1.0 ml/min. Compounds were detected at a wavelength of 445 nm.

### Color

The color distribution of mango chips was described by a new approach developed specially for this study (21). Therefore, a detailed description will be given. Four pieces of mango chips from each frying experiment were photographed and analyzed via a color quantization for each pixel based on a method described by Wu (22).

Images of mango chips were taken using a color digital camera (Canon 1000D with Canon EFS 18-55 mm F3.5-5.6 IS lens) mounted on Kaiser RT1 base 25 cm from the product and were placed inside a closed picture chamber. The light used was produced by a 4 × 36 watt 5,400 k 40 Hz fluorescent light mounted at 22° from the sample axis. Color calibration was done using X-Rite ColorChecker Passport and Adobe Lightroom.

An uncompressed picture file (CR2) with image size 3,888 × 2,592 pixel was produced and later converted to another uncompressed file (Exif-tiff 8 bit) at the same resolution by image processing software (Canon Digital Photo Professional, version 3.14.40.0) prior to further analysis. Image background was removed using a quick selection tool from an image processing software (Adobe Photoshop CC 2015). The tiff images were then analyzed using Color Inspector 3D v 2.3(21) within Fiji (22), an Image J 1.52 g repository (23), according to color quantization for each pixel. The obtained RGB color table was converted into L^*^ a^*^ b^*^ values using the method developed by (24). Only the L^*^ and a^*^ values were used, as they describe the most important changes in color of vacuum-fried products (6). The L^*^ value describes the lightness with 0 for darkest black and 100 for brightest white and was divided into 3 levels, 0 ≤ dark ≤ 60, 60 < medium-light ≤ 80, and 80 < light ≤ 100. The a^*^ value describes the transition from green to red, ranging from −128 for the purest green and +128 for the purest red, and were divided into 3 levels, green ≤ 0, 0 < medium-red ≤ 10, and red >10. The number of pixel in each lightness and redness level is then divided by total pixel of the mango chips and multiplied by 100% to produce % of pixels of each lightness and redness level.

### Statistical Analysis

Data analysis was performed using R software by independent *T*-Test for TSS and firmness to test the difference between ripe and unripe mango. On the other hand, ANOVA with Tukey *post hoc* analysis was performed for moisture, fat, texture, TAA, β-carotene, and color. Numbers and graphs were made based on mean and standard error. Standard error of the mean (SEM) was used to describes the uncertainty of how the sample mean represents the population mean, which is possible at a large number of measurements (23).

## Results and Discussion

### Firmness and TSS of Fresh/Raw Mango

Firmness and TSS content of raw mangoes were measured to determine the required ripeness stages (Table 2). Commonly during mango ripening, the TSS content increases due to hydrolysis of starch into sugars (14), while firmness of the fruits declines due to breakdown of the cell wall polysaccharide such as pectin (24). However, our data just show just a slight though significant increase in TSS from unripe to ripe mango (0.38 °Brix). While Ibarra-Garza and Ramos-Parra (14) found a higher increase during ripening in Keitt mango, from 10.1 to 12.7 °Brix. This difference could be ascribed by variation among varieties.

### Moisture Content

**Figure 1:**
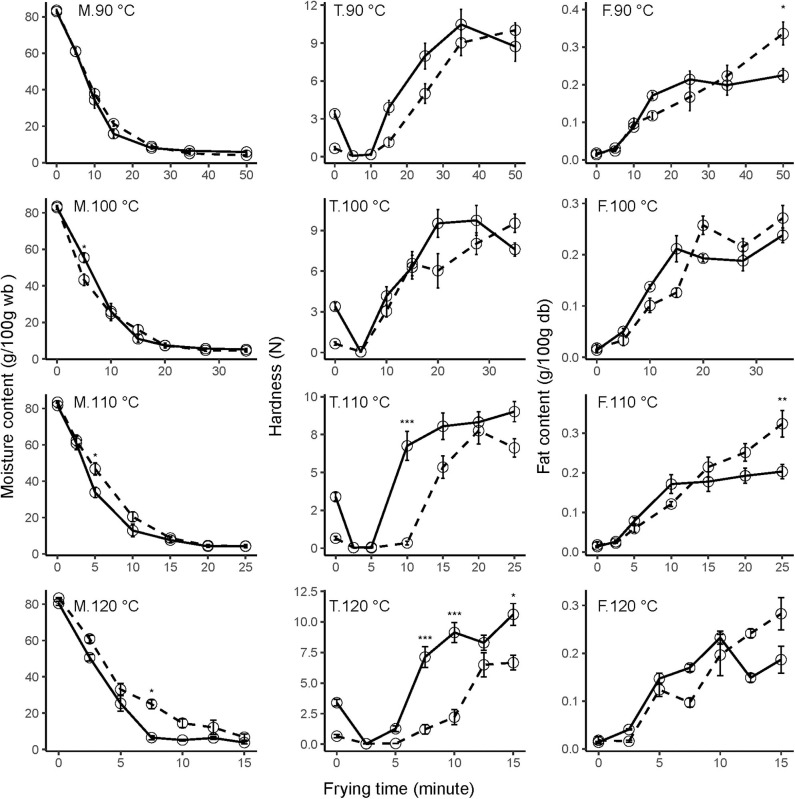
Effect of frying temperatures, time, and ripening stage on moisture content (M), texture (T), and fat content (F) of mango chips during vacuum frying at 90, 100, 110, and 120°C. Solid lines are unripe mango, and dashed lines are ripe mango. Error bars are standard error. Asterisk shows significant difference between ripening stages (*p ≤ 0.05, **p ≤ 0.01, **p ≤ 0.001, ***p ≤ 0.0001). *N* = 6 (two frying experiments × 3 replications) for moisture content; 4 (two frying experiments × 2 replications) for fat content; 20 (two frying experiments × 10 replications) for texture.

Overall, moisture loss during vacuum frying exhibited a classical drying profile (Figure 1). As frying time increases, moisture content rapidly decreases. For all temperatures, an initial rapid decrease in water content was observed, followed by a more gradual decline until a constant moisture value is reached. Moreover, moisture loss increased with increasing temperature; consequently, a shorter frying time led to the same final moisture content.

At 10 min of frying, the moisture content of unripe mango ranged from 34.4 ± 4.4% at 90°C to 5.2 ± 0.5% at 120°C, while for ripe mango the content was 37.8 ± 2.9% and 14.5 ± 2.4% for the respective temperatures. Even though not significant, this result shows that the moisture of ripe mango was more difficult to evaporate during frying than for unripe mango. This difference could be explained by the structure, soluble solids, and texture differences. Pectin is an important compound influencing those physical characteristics. Pectin is more abundantly present in unripe mango, thereby increasing the water-binding properties (25). In unripe mango, large pectin molecules firmly hold the water and, when heated, the polysaccharides shrink expelling water out and increase the moisture loss (26). On the other hand, in the ripened fruit the water is already more free because pectin was enzymatically hydrolyzed.

Moreira (1) described that a moisture content of <10% is needed to keep the product stable upon storage. To produce fried chips with <10% moisture content, vacuum frying at 120°C will need 7.5 min for unripe mango compared to 15 min that is required for ripe mango. A similar effect was observed for air-drying of 1-cm-thick banana slices, for which the rate constant of ripe banana was lower than in unripe banana, even though the difference was not significant (27).

### Fat Content

During vacuum frying, there is an increase in the fat content for mango parts of both ripening stages; however, the fat content of unripe mango showed a sigmoid trend, while ripe mango showed an almost linear increase in fat content during frying (Figure 1). A similar increase in fat content is also observed in vacuum frying of apple (5).

Fat uptake during vacuum frying is a direct effect of moisture loss. When the mango slices were submerged in hot oil, moisture rapidly evaporated from the surface and allowed oil to adhere to the dry surface (28) and further infiltrate to the chips (29).

At most frying conditions, fat content in unripe and ripe mango shows no significant differences, except at the most intense treatments in which the fat content in unripe mango is lower as shown at 90°C for 50 min (*p* = 0.0150) and 110°C for 25 min (*p* = 0.0034), but at 120°C, there was no significant difference for all frying times. The difference could be caused by the higher pectin content in unripe mango; BeMiller (30) describes that pectin consists of hydrophilic and hydrophobic molecules. Pectin could become a barrier to oil absorption (31).

### Texture

After vacuum frying started, the hardness of fried mango chips initially drops for both ripening stages and at all temperatures and subsequently increases in time. Dueik and Robert (32) divided the vacuum frying into fast and slow phases. During the fast phase, the plant tissue initially softens and then hardens in the slow phase. The tissue softens because the middle lamella between the cells is solubilized (33). However, during the slow phase there is tissue hardening because the mango dehydrates and forms a crust.

The hardening process accelerates at higher temperatures. Unripe mango chips fried for 15 min at 90°C had a hardness value of 3.9 ± 0.6 N; this value was at the beginning of the slow phase which increased at a longer frying time. On the other hand, unripe mango chips fried at 120°C for 15 min had a hardness value of 10.6 ± 0.9 N; this value was at the end of the slow phase. Nunes and Moreira (13) did a similar research on Tommy Atkins mango chips and found that when the oil temperature was increased from 120 to 130°C, the maximum force (N) and the work (N ^*^ mm) values also increased. However, when the temperature was increased to 138°C, these maximum force and work values decreased because of the brittleness of the chips. A phenomenon was also observed in our data for unripe mango fried at 90 and 100°C and ripe mango fried at 110°C.

Overall, there was no significant difference between hardness of unripe and ripe mango after vacuum frying at 90 and 100°C at all-time points. At higher temperatures, the hardening was faster for unripe mango compared to ripe mango. A significant difference (*p* = 0.000) is observed after frying at 110°C for 10 min, with a hardness of 6.8 ± 1.0 N for unripe mango and 0.3 ± 0.1 N for ripe mango. When the temperature increased to 120°C, the difference (*p* = 0.000) was observed at a shorter frying time (7.5 min): 7.1 ± 0.9 N for unripe mango, and 1.2 ± 0.4 N for ripe mango. Again, the difference in matrix could play a role here. The crust formed by unripe fried mango is harder because the pectin polymer is still there, and when the water evaporates, it is able to form a stronger network and becomes hard (34).

### Ascorbic Acid

Vitamin C (AA + DHA) is an important nutritional parameter for fried food products. The raw material used in this study is characterized by a high total ascorbic acid content; raw unripe mango had a higher TAA content of 156.2 ± 6.4 mg /100 g compared to ripe mango (126.6 ± 3.0 mg/100 g). The TAA decrease could be a result of ascorbate peroxidase (APX) activity which uses ascorbates as electron donor to remove hydroxyl radical from the cell that produced during fruit ripening (35).

**Figure 2:**
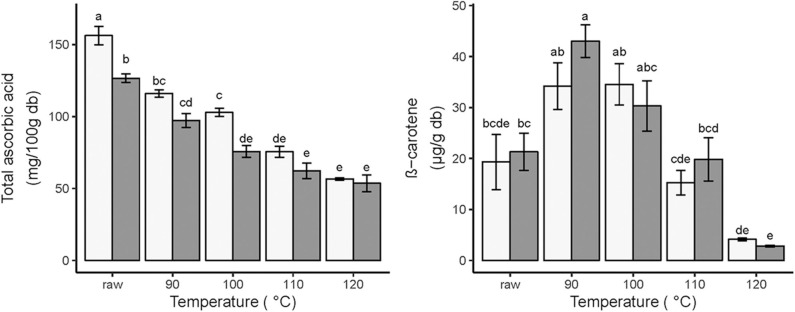
Effects of vacuum frying and ripening stages of mango on total ascorbic acid (mg/100 g db) (left) and β-carotene (μg/g db) (right) during vacuum frying. Light bars represent unripe mango; dark bars represent ripe mango. The different frying temperatures also represent the different final frying times; 90°C (50 min); 100°C (35 min); 110°C (25 min); 120°C (15 min). Error bars are standard error. Different letters above the error bars show significant difference between treatments *N* = 6.

As expected, thermal degradation of total ascorbic acid (TAA) increased with increasing frying temperature; a similar pattern was observed in both ripening stages (Figure 2). AA loss was described to have a linear relationship with temperature in vacuum-fried gold kiwi fruit (36) but also by first-order kinetics and the Arrhenius equation (37). Reduction in ascorbic acid content (AA) is possible in the absence of oxygen and at relatively low frying temperatures as it can follow an anaerobic pathway of non-enzymatic browning reactions (5). It has been shown in apple and potato that frying at atmospheric pressures substantially reduces the vitamin C content in comparison with vacuum-frying conditions (4). Under vacuum pressure, no dehydroascorbic acid is formed in significant amounts; ascorbic acid degrades by the cleavage of ring and the addition of water, decarboxylation, and intermolecular rearrangement, followed by dehydrations to produce furfural (38).

However, the TAA values remain higher in the unripe mango for all temperatures, although only significantly at 100°C. Frying for 35 min at 100°C retained 65.8 and 59.8% of TAA in unripe and ripe mango. Even at this severe heating, 100 g unripe and ripe vacuum-fried mango is able to provide 114.3 and 84.1% of the recommended daily allowance of vitamin C for adults (90 mg), respectively. This difference shows the importance of the fruit matrix as the container of the ascorbic acid. Davey et al. (39) mentioned that L-ascorbic acid was present at the subcellular level in various cell compartments including in chloroplast, cytoplasm, mitochondria, and apoplast. This arrangement could protect ascorbic acid inside the cell, but due to the lower amount of pectin in the cell walls of the ripe mango cell (34) could increase ascorbic acid heat damage in comparison to unripe mango cells.

### β-Carotene

Raw ripe mango had a higher β-carotene content compared to unripe mango, even though not significant (27.2 ± 3.6 and 19.3 ± 5.4 μg/g db, respectively). This result was expected since carotenoid content increases during mango ripening (14).

There was no significant difference in β-carotene content for each temperature/time combination between the two ripening stages. While in both ripening stages the initial low β-carotene value increases after frying at 90 and 100°C, there is a clear decline in β-carotene content for both ripening stages when temperature is increased (Figure 2).

The possible explanations for the increased ß-carotene concentrations after frying at 90 and 100°C, which were also found in vacuum-fried apricot at 70–90°C (40), could be connected to the role of the changing fruit matrix on the accessibility of ß-carotene. In mango, β-carotene is located in lipid-dissolved and liquid-crystalline tubular elements of mesocarp chloroplasts. Thermal treatment and the presence of lipids improve the β-carotene accessibility (41).

However, after vacuum frying at 110 and 120°C, the ß-carotene concentrations decreased, which shows the thermal sensitivity of ß-carotene in vacuum frying. Da Silva and Moreira (12) confirm that vacuum frying of mango at 1.33 kPa, 120°C, for 3 min resulted in a decrease by 71.3% of the ß-carotene content. So, it was clear that to maintain ß-carotene content in vacuum-fried mango chips, a high-temperature processing should be avoided.

### Color

**Figure 3:**
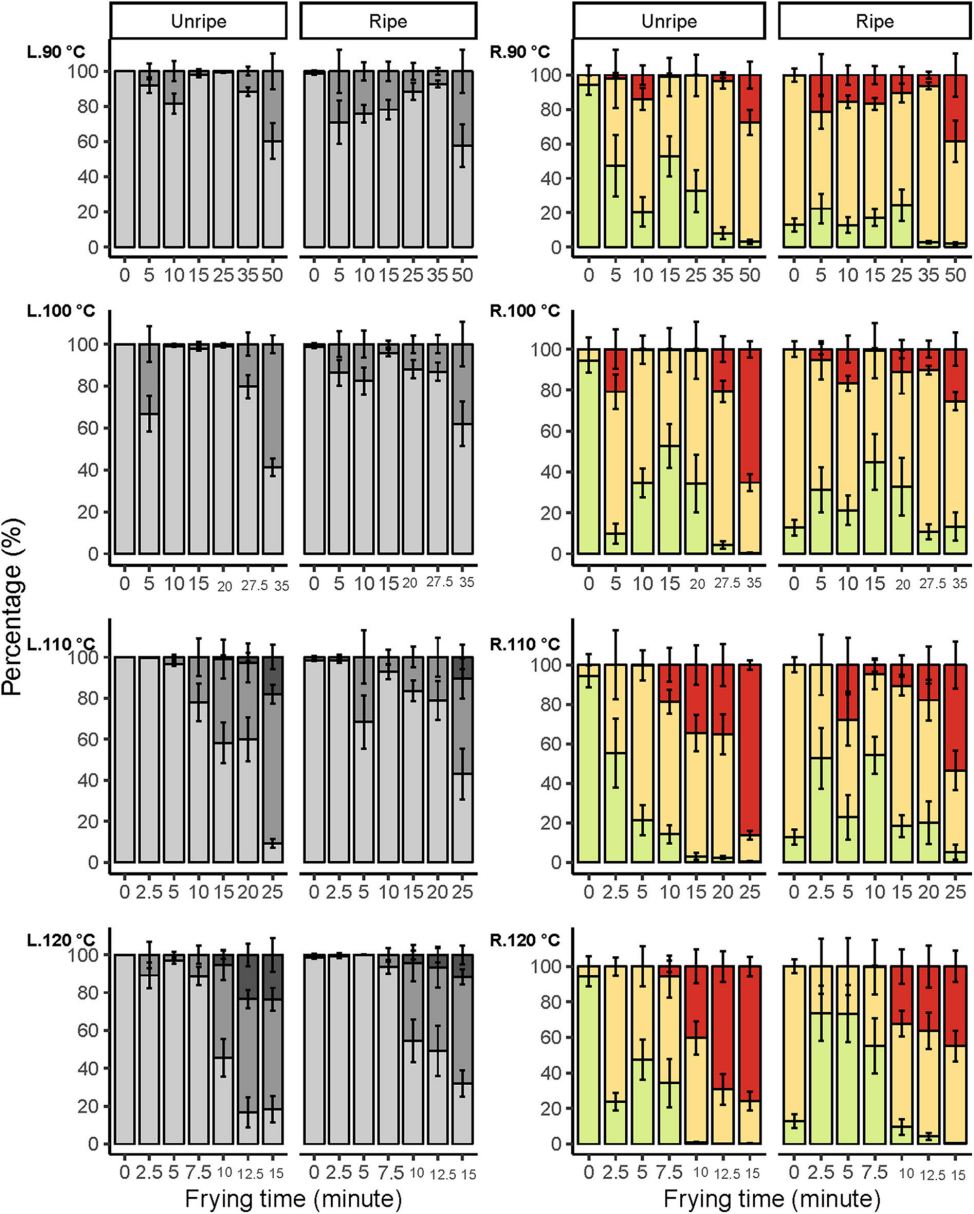
Color distribution in terms of lightness L* (L) and green to red a* (R) changes during vacuum frying at different ripening stages of mango, fried at 90, 100, 110, and 120°C. Lightness levels of mango were represented in three levels: 0 ≤ dark ≤ 60, 60 < medium-light ≤ 80, 80 < light ≤ 100. Meanwhile, red to green were represented in three levels; green ≤ 0, 0 < medium-red ≤ 10, red > 10. Error bars are standard error *N* = 8.

**Figure 4:**
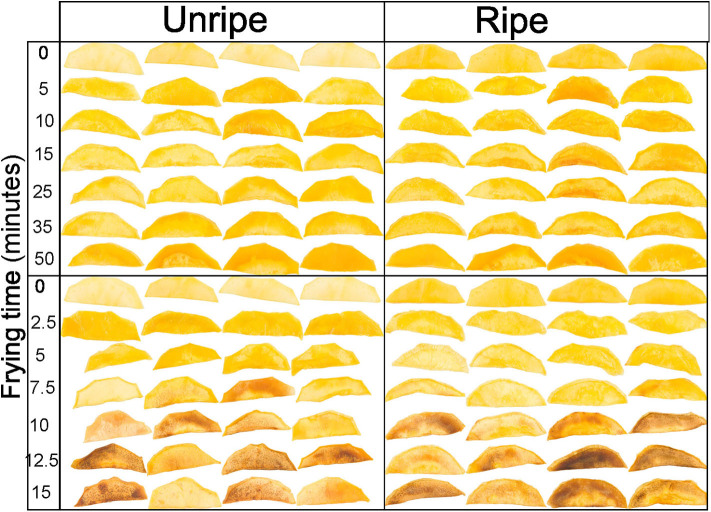
Unripe (left) and ripe (right) mango slices vacuum fried at 90 (top) and 120°C (bottom) for different times.

The color of fried mango chips is often inhomogeneous with strong local differences, e.g., the occurrence of brown spots in a lighter background. This phenomenon is present in most fried or baked foods. Therefore, measuring the overall color changes, e.g., expressed as L^*^a^*^b^*^ values, is not representative for the visual appearance. In many cases, the food matrix and surface are not homogenous and have different structures at micro- and macroscopic scales (42). Image analysis of photographs taken under standard lighting gives the opportunity to assess the local differences in color values in a quantitative way. Instrumental color distribution analysis has been done for food for a variety of applications, including for microwaved pizza (43); however, application of this method in vacuum-fried food and especially as a time-series analysis is a novel approach. The effects of vacuum frying and ripening stages on the color changes have been assessed in terms of the percentage of surface area in levels of lightness (L^*^) and green to red (a^*^) (Figures 3, 4).

The area in levels of lightness decreased gradually upon increasing frying time and temperature. However, a faster trend in lightness reduction was observed for the unripe mango chips, which was most distinct at 110°C and 120°C. The reduction in levels of the light area clearly led to an increase in levels of medium-light and dark areas, also differentiating between the two maturities. Similar results were found by Maity and Bawa (44), which state that vacuum-fried jackfruit bulb at 90°C for 25 min could reduce lightness by 15% and, when extended for another 5 min, reduced to 32%.

Similarly, the areas for medium-red and red increased progressively as the frying time and temperature increased, which was also observed by Maity and Bawa (44), stating that vacuum-fried jackfruit bulb at 90°C for 10 min increased the a^*^ value by 168% and by 303% when extended to 20 min of frying. Furthermore, when fried at 100°C, the a^*^ value was increased to 367% after 20 min, whereby a faster and stronger increase in redness areas was observed for unripe compared with ripe mango chips. At all temperatures, the green area decreased in unripe mango, while a fluctuation was seen in ripe mango chips. This trend was most pronounced at the highest temperatures. Additionally, as water evaporated, the boiling point of the moisture in the fruit will be increased and so will the fruit temperature (41), which indirectly contributed to color changes.

It was clear that the combination of ripening stage, frying time, and temperature has a substantial influence on the color of the mango chips. Unripe mango fried for a longer time at a higher temperature has a darker and redder surface compared to ripe mango. So, unripe mango seems more susceptible to the temperature–time treatments toward changes in lightness and redness compared to ripe mango. Similar effects were also found in apple, although they were measured as having an average color value of the fried apple surface (28). The change in color at 110 and 120°C at different frying times is probably due to the Maillard reaction and/or caramelization. The darker areas (Figure 4), and sometimes spots, would then be caused by locally higher amounts of reducing sugars, which could increase the rate of both the Maillard reaction and caramelization (40). Caramelization is likely to occur due to the high amount of sugars (45), also confirmed by (46) in vacuum-fried jackfruit. However, ripe mango has a significantly higher sugar content (Table 2), so the darker color was expected to be dominating, but this is not the case; frying time and temperature and other mechanisms could play more roles on the color change.

## Conclusion

Moisture loss of unripe mango chips was faster than that of ripe mango chips. There was no significant difference between hardness of unripe and ripe mango after vacuum frying at low temperatures (90–100°C), but at higher temperatures (110–120°C), unripe mango had a higher hardness value compared to ripe mango. Vacuum-fried ripe mango had a higher fat content compared to unripe mango. No differences between the ripening stages were found on the degradation of ascorbic acid and β-carotene during frying. Unripe mango is more susceptible to temperature and time toward lightness and redness changes compared to ripe mango. Considering all quality parameters, unripe mango is preferred over ripe mango for vacuum-frying processing. Furthermore, vacuum frying at 100°C for 20 min was sufficient to decrease the moisture content and produce high-hardness chips without adsorbing too much oil, maintain the color without losing too much ascorbic acid, and preserve the β-carotene content.

## Data Availability Statement

The raw data supporting the conclusions of this article will be made available by the authors, without undue reservation.

## Author Contributions

FA did the study conception and design, acquisition of data, analysis and interpretation of data, and drafting of the manuscript. EG and JV did the study, acquisition of data, analysis and interpretation of data, and drafting of the manuscript. MD did the analysis and interpretation of data, study conception and design, drafting of the manuscript, and critical revision. VF did the drafting of manuscript and critical revision. RV did the study conception and design, drafting of the manuscript, and critical revision. All authors contributed to manuscript revision and read and approved the submitted version.

## Conflict of Interest

The authors declare that the research was conducted in the absence of any commercial or financial relationships that could be construed as a potential conflict of interest.
